# Pharmaceutical Processes

**Published:** 1800-06

**Authors:** 


### PHARMACEUTICAL PROCESSES.

Citizen Fourcroy relates a new method of combining the oxygen with ſuet, to make the oxygenated pomatum. Take of purified lard as much as you like; let it melt by a gentle fire in an earthen veſſel, add to it afterwards two-thirds its weight of pure nitric acid 28—30 degrees ſtrong, and ſtir the mixture with a wooden ſpoon, till it be cool; then put the whole into thirty times its weight of rain or river water, and let it boil for half an hour, and ſtir it continually, till it becomes cool. After having ſeparated the fat from the water, melt it again, and preſerve it in a glaſs or earthen veſſel. Alyon's method is ſomewhat different from this; he takes ſixteen parts of purified lard, and one part of nitric acid, 32 degrees ſtrong. When the fat is melting, he adds the acid, and ſtirs it with a glaſs tube, by which means the azote is deſtroyed during the boiling, and the oxygen only remains combined with the ſuet, and he does not put it in water to purify. The fat imbibes in this manner more oxygen, and it is therefore a queſtion which method is preferable. The lard treated after Fourcroy's preſcription contains but one-third of its weight of oxygen, and Alyon's almoſt double the quantity. Fourcroy remarks, that if the oxygenated pomatum ſhould not prove of any avail in medicine, it might be made uſe of to ſubdue quickſilver the ſooner, and to prepare the ointment of it in a fifth of the time that is uſually required. *Tromſdorf's Journal der Pharmacie, Vol. viii. No. i. p.* 162.

---

The ſame chemiſt lately diſcovered that the Kermes mineral and the ſulphur auratum antimonii contained ſulphurated hydrogen (gas hydrogéne ſulfuré) in their mixture. The difference between them conſiſts in the former only containing ſulphurated hydrogenous oxyd of antimony, and the latter alſo has ſulphurated oxyd of antimony. The diaphoretic and ſometimes vehement action of the Kermes mineral, ſeems undoubtedly to depend upon the hydrogéne ſulphuré which is combined with it.

It

It would be worth while to examine, by an accurate analysis, the proportion of the hydrogen, sulphur, and oxyd of antimony in the Kermes, according to the different modes of preparing it, for the purpose of knowing whether it would not be better to prepare it by the simple combination of an oxyd of antimony, and of water saturated with gas hydrogené sulfuré. *Ibid. p.* 106.

---

To preserve extracts, particularly the bitter ones, which contain more gummy than resinous parts, from being spoiled when kept for some time, Citizen Demachy proposes to add the eighth part of alkohol when the extract begins to thicken, and to continue the evaporation afterwards. He is of opinion that the alkohol has that effect by being partly a mean of affinity between the gummi and resina, partly because it destroys the mucilaginous parts inherent to some resins, and produces thereby a more simple substance. *Ibid. p.* 181.

---

Citizen De Launay proposes an easier method of making mercurial ointments, viz. to rub the quicksilver with some old sweet oil, which has already become a little rancid; one drachm of this is sufficient to subdue a pound of mercury in a very short time: The lard is afterwards added by degrees. *Ibid. No. ii. p.* 27.

---

Mr. Juch proposes a new method of preparing the muriated barytes, or terra ponderosa salita, worthy the attention of apothecaries and druggists, which is as follows: Take one part of finely pulverized barytes or heavy spar; after having burned it, and quenched it in water, add two parts and a half of pot-ash. This mixture is melted, and kept on the fire for an hour and a half, in an earthen vessel, of which those fabricated in Hessi are particularly famous among the German chemists. When the whole mass is entirely fluid, pour it into a clean iron kettle, and boil it well with common water for the sake of clearing it from the sulphate of pot-ash or vitriolated tartar. The barytes obtained in this manner is still mixed with some undissolved spar. Saturate it now perfectly with muriatic acid, and let it evaporate. The remaining dry mass of salt is melted again in an earthen vessel; and being in a quiet state of fluidity, pour it upon a stone plate, and cover it with a vessel, to prevent any thing escaping, which sometimes happens in cooling. To give this mass a fine, regular crystallization, and to separate it from other heterogeneous matters, it must be dissolved in a sufficient quantity of distilled boiling water. It is not necessary to pulverize it before, because a greater part remains undiſſolved, when pulverized, than when the whole is put into the veſſel. In this ſolution, volatile liver of ſulphur, diſſolved in water, is dropped as long as any precipitation is viſible; by which means it is cleared from any adherent metallic particles. The fluid is now filtered, and gently evaporated to the point of cryſtallization. *Tromsdorf's Journal der Pharmacie, Vol. vii. No. ii. p.* 27.

---

Mr. Eacard, of Bamberg, has found the following method of preparing a tinctura opii, much cheaper than laudanum liquidum, and which does not ſo eaſily precipitate as this, and wherein the quantity of opium is more accurately determined. ℞. opii optim. unc. ii. caryophyllorum dr. i. aqu. cinnamom. unc. viii. alcohol vini unc. iv. opii caryophyllis in pulverem tritis aqua cinnamomi cum alcohol permixta affunditur, vitrum bene clauditur. Digeſtione per vi dies in loco calido continuata, tinctura exprimitur clarificatur.—Ten grains of this tincture contain one grain of opium, if good opium has been employed. *Examination of the Brunonia System by Experiments, No. iii. p.* 105.

---

A new preparation of mercury has been invented at Petersburgh, called the Mercurial Soap, which is ſaid to be of very great avail in obſtinate venereal complaints. A ſolution of quickſilver in diluted nitrous acid, or aqua fortis, is mixed with a ſolution of white Spaniſh ſoap; whereby an oily ſubſtance riſes on the ſurface of it, which forms with cauſtic alkali a ſoap containing mercury. Two ſcruples of this remedy are diſſolved in two ounces of diſtilled water, and it is given in drops. Hufeland gave it to eighty drops twice a day, with ſome ſucceſs. *Hufeland's Journal, Vol. v. No. iii.*

---

Cit. Lartique propoſes a method of preparing tartar emetic by combining the cream of tartar with the grey oxyd of antimony. The preparation thus obtained, has the preference of having a certain and more equal ſtrength than tartar emetic obtained in the common way. *Journal des Pharmaciens, No. x. p.* 122.

---

Mr. Gräff, at Bareuth, made ſeveral experiments to obtain ſugar from different vegetable ſubſtances. All ſpecies of gramineous plants contain a conſiderable quantity of ſugar when young. The Arunde Phragmitis, Lin. gave in 16 lb. three ounces of ſugar. Parſnips are alſo very productive of ſugar. *Tromsdorf's Pharmaceutical Journal,* 1800.

---

*Discovery*

